# Emotional interference and right prefrontal hemodynamic responses in adult cannabis users: a pilot fNIRS study

**DOI:** 10.3389/fpsyg.2026.1873912

**Published:** 2026-07-24

**Authors:** Sergio Castaño Castaño, Alexandre Díaz-Pons, Ángel Yorca-Ruiz, Víctor Ortiz-García de la Foz, Juan Luis Martín Ayala, Angel Olider Rojas Vistorte, Rosa Ayesa-Arriola

**Affiliations:** 1 Department of Psychobiology, Faculty of Health Sciences, University of Burgos, Burgos, Spain; 2 Mental Health Research Department, Marqués de Valdecilla Research Institute, Santander, Spain; 3 Faculty of Health Sciences, Universidad Europea del Atlántico, Santander, Spain

**Keywords:** cannabis use, cognitive control, emotional interference, Emotional Stroop, fNIRS, prefrontal hemodynamics

## Abstract

**Introduction:**

Cannabis use has been linked to alterations in cognitive control and emotional processing, although findings vary according to use pattern and abstinence status. This pilot study examined whether emotional interference is especially sensitive to short-term abstinence-related differences across cannabis-use profiles and whether such differences are accompanied by distinct prefrontal hemodynamic responses.

**Methods:**

Non-users, occasional cannabis users, and chronic cannabis users completed a pre–post cognitive battery including an Emotional Stroop task. In the cannabis-use groups, the post session followed 72 h of abstinence. Behavioral outcomes were analyzed using non-parametric tests. Prefrontal oxygenated and deoxygenated hemoglobin responses were recorded with functional near-infrared spectroscopy (fNIRS) and analyzed using mixed-effects models with false-discovery-rate correction, complemented by Bayesian contrasts on post–pre change scores.

**Results:**

Behavioral effects were concentrated in the Emotional Stroop task. Occasional users showed a significant pre–post reduction in response latency for negative stimuli, together with an accuracy-related pattern in between-group comparisons. Chronic users showed no significant behavioral changes, whereas controls showed a latency change compatible with possible retest effects. fNIRS analyses revealed a significant time × group interaction for deoxygenated hemoglobin in right and bilateral prefrontal regions.

**Discussion:**

These preliminary findings suggest that emotional interference may be more sensitive than non-emotional cognitive performance to short-term abstinence-related differences across cannabis-use patterns.

## 1 Introduction

Cannabis use has been associated with alterations in cognitive control, emotional processing, and self-regulatory functions, although the magnitude, direction, and persistence of these effects vary considerably across studies. Such heterogeneity appears to depend on multiple factors, including pattern of use, abstinence status, and the cognitive or affective demands imposed by the task. Recent reviews suggest that cannabis-related differences are often modest and not uniformly expressed across cognitive domains, raising the possibility that emotionally salient paradigms may be more informative than non-emotional tasks for detecting subtle functional variability. In particular, tasks requiring cognitive control under affective distraction may provide a useful window into cannabis-related differences in emotion–cognition interaction, especially when distinct consumption profiles are compared across repeated assessments. From this perspective, within-subject pre–post approaches combining behavioral and neurophysiological measures may be especially useful in pilot studies aimed at identifying preliminary neurofunctional correlates rather than demonstrating broad, generalized impairment (Broyd et al., 2016; Dellazizzo et al., 2022).

A plausible neurobiological basis for this approach lies in the effects of Δ9-tetrahydrocannabinol (THC), a partial CB1 receptor agonist, on fronto-limbic circuitry involved in emotion regulation and executive control. Functional neuroimaging studies indicate that THC can modulate amygdala responses to threat-related cues and alter functional coupling between limbic regions and prefrontal areas such as the rostral anterior cingulate cortex and dorsolateral prefrontal cortex. These effects are relevant because paradigms that integrate emotion and cognition, such as the Emotional Stroop, require participants to suppress emotionally salient information while maintaining goal-directed responding. Accordingly, cannabis-related differences may become more apparent under conditions of emotional interference than in “cold” cognitive tasks that do not strongly recruit affective regulatory systems (Phan et al., 2008; Gorka et al., 2014; Gorka et al., 2016).

Within this context, functional near-infrared spectroscopy (fNIRS) offers a practical method for quantifying cortical hemodynamic responses, including oxygenated and deoxygenated hemoglobin, with good tolerance to movement and relatively high ecological portability. fNIRS is particularly well suited to monitoring prefrontal dynamics during tasks involving executive control and emotion–cognition interaction, and methodological advances have strengthened its use in cognitive and clinical neuroscience, including improved recommendations for preprocessing, artifact handling, and physiological interpretation. Importantly, previous work has shown that acute THC exposure can modulate prefrontal hemodynamic responses measured with fNIRS during working memory and cognitive control tasks, with increases in prefrontal HbO varying as a function of intoxication and task load. However, evidence remains limited for studies that combine repeated behavioral assessment with concurrent prefrontal fNIRS during affect-laden interference paradigms while also contrasting different patterns of cannabis use (Ferrari and Quaresima, 2012; Scholkmann et al., 2014; Bendall et al., 2016; Pinti et al., 2020; Keles et al., 2017; Gilman et al., 2019).

Several gaps therefore motivate the present study. First, most prior cannabis–fNIRS studies have examined acute intoxication rather than short-term abstinence, leaving it unclear whether emotional interference effects persist beyond the acute phase and whether they differ according to use frequency. Second, occasional and chronic users are often pooled or compared only cross-sectionally, limiting the ability to determine whether distinct cannabis-use profiles show different behavioral and prefrontal hemodynamic changes over time. Third, it remains unknown whether emotional interference yields more consistent effects than non-emotional tasks, such as the classic Stroop, CPT, or Symbol Search, within the same individuals across repeated assessments. Clarifying these points is important because single-session designs may obscure use-pattern-specific differences that emerge only when recent exposure status is standardized and behavior is examined under affective challenge.

In this pilot proof-of-concept study, we combined behavioral assessment and prefrontal fNIRS in a pre–post design including non-users, occasional cannabis users, and chronic cannabis users. The primary aim was to examine whether emotional interference was more sensitive than non-emotional cognitive performance for detecting short-term abstinence-related differences across cannabis-use profiles. A secondary aim was to determine whether any such behavioral differences were accompanied by distinct prefrontal hemodynamic responses during task performance. By integrating behavioral and cortical hemodynamic measures within an Emotional Stroop paradigm, the study addresses how cannabis-use patterns may relate to cognitive-affective control and its prefrontal functional correlates. We expected occasional users to show a more pronounced pre–post shift than controls, together with differential prefrontal modulation, whereas chronic users were expected to show a different profile potentially compatible with functional adaptation and/or altered processing efficiency. Given the small sample size and exploratory nature of the study, these expectations were treated as preliminary and hypothesis-generating.

## 2 Methods

### 2.1 Study design

This pilot, exploratory proof-of-concept study used a comparative pre–post design comprising three groups defined by cannabis-use pattern: non-consumers (controls), occasional users, and frequent/chronic users (total *N* = 27; *n* = 9 per group). Participants completed four computerized cognitive tasks (Symbol Search, Continuous Performance Test [CPT], Stroop, and Emotional Stroop) at two time points (PRE and POST). In the cannabis-use groups, the POST assessment took place after a minimum of 72 h of abstinence to standardize recent exposure status prior to reassessment. This design allowed the characterization of within-subject change over time and between-group differences across cannabis-use profiles while participants performed the same cognitive battery under concurrent physiological monitoring.

### 2.2 Participants

Participants were adults (≥18 years) recruited through cannabis user associations, university advertisements, and social media. Based on self-reported cannabis-use status, participants were assigned to one of three groups: frequent/chronic cannabis users (CAN-F), defined as individuals reporting ≥10 cannabis-use episodes in the last month; occasional cannabis users (CAN-S), defined as individuals reporting <10 cannabis-use episodes in the last month; and non-consumers (NC), defined as individuals reporting never having used cannabis.

For both cannabis-use groups, a minimum 72-h abstinence period prior to the second assessment session was required and verified by self-report. Assessments were conducted between 09:00 and 19:00, depending on participant availability, during the period from October 21, 2024 to January 28, 2025. The rationale for the ≥72-h abstinence interval is described in Section 2.3.

Inclusion criteria were normal or corrected-to-normal vision and absence of neurological or psychiatric disorders. Exclusion criteria were current psychopharmacological treatment, pregnancy, or dermatological conditions that could interfere with placement of the fNIRS cap. Sociodemographic information was collected via structured interview and included age, sex, education level, employment/student status, annual income, and residential setting, classified as urban (>10,000 inhabitants), intermediate, or rural (<2,000 inhabitants).

The structured interview also explored clinical history, psychiatric or neurological diagnoses, potential comorbidities, current psychotropic medication use, relevant physical symptoms, and substance-use characteristics. For cannabis users, additional information was collected, when available, regarding use frequency, pattern of use, and abstinence-related symptoms or behaviors prior to the POST assessment. Information on alcohol, nicotine/tobacco, and other psychoactive substance use was also explored descriptively when reported by participants, given their potential relevance as confounding factors in cognitive and hemodynamic measures.

Given the small size of this pilot sample, participant-level sociodemographic, clinical, and substance-use details are not reported in a highly granular manner in order to preserve anonymity and reduce the risk of indirect identification. These variables were inspected descriptively to characterize the sample, verify eligibility, and contextualize the interpretation of the findings. However, they were not included as covariates in the inferential analyses because of the limited sample size and the risk of unstable model estimates. Limitations related to sample size, self-reported cannabis exposure, abstinence verification, and unmeasured substance-use heterogeneity are addressed further in the Limitations section.

### 2.3 Rationale for the ≥72-h abstinence requirement

A minimum abstinence period of ≥72 h prior to the POST session was implemented to reduce variability due to very recent intoxication and to standardize participants’ recent consumption state across users. Conceptually, this interval was chosen to enable assessment of residual and/or partially recovering effects following short-term abstinence, rather than acute intoxication effects. This approach is consistent with prior work suggesting that cognitive/affective performance and prefrontal responses may remain altered beyond the immediate acute window, while also allowing for partial normalization over the first days of abstinence (Scott et al., 2018).

### 2.4 Neurocognitive tasks

All participants completed the same computerized cognitive battery in a fixed order: classic Stroop, Symbol Search, Emotional Stroop, and Continuous Performance Task (CPT). Stimuli were presented on a 23.8-inch monitor at an approximate viewing distance of 70 cm, while prefrontal fNIRS was recorded continuously throughout task performance. Before each task, participants received standardized on-screen instructions and completed a brief practice phase to ensure comprehension of the response rules. The only exception was the CPT, which began with a nine-digit visual countdown presented over the task background to facilitate visual adaptation before the start of the experimental trials.

The fixed task order was maintained across participants to preserve consistency between the PRE and POST sessions and to minimize procedural variability in this pilot pre–post design. Between tasks, participants viewed on-screen instructions and had an interval of approximately 1 min before beginning the next task. For all tasks, trial-level behavioral data were recorded, including response accuracy and reaction time when applicable. The same task parameters, response mappings, randomization rules, and acquisition procedures were used at PRE and POST.

### 2.5 Stroop task (cognitive interference)

The classic Stroop task was used as a non-emotional measure of cognitive interference. On each trial, an English color word (“red,” “green,” “blue,” or “yellow”) was displayed in colored ink. Participants were instructed to identify the ink color while ignoring the semantic meaning of the word. Responses were made using four arrow keys with a fixed mapping: left = red, right = green, up = blue, and down = yellow. Stimulus text height was set to 0.1 in normalized display units.

Word identity and ink color were selected independently at random, with the restriction that the same ink color could not be repeated on consecutive trials. This procedure yielded an approximate distribution of 25% congruent trials, in which word meaning and ink color matched, and 75% incongruent trials, in which they differed. The exact trial sequence could therefore vary across participants while preserving the same overall task structure. Each stimulus remained on screen until the participant responded, after which a blank screen was presented for 1,000 ms before the next trial.

Participants completed 10 practice trials before the experimental phase. The test phase consisted of 100 trials. For each trial, the program recorded the word, ink color, congruency condition, response accuracy, and reaction time in milliseconds. Behavioral outcomes included overall accuracy, overall mean reaction time, mean reaction time stratified by response accuracy, and Stroop interference, defined as the reaction-time difference between incongruent and congruent trials.

### 2.6 Symbol search (processing speed and visual search decision-making)

The Symbol Search task was used as a non-emotional measure of processing speed, visual scanning, and decision-making. Each self-paced trial displayed two target symbols at the top of the screen and five search symbols below. Symbols were presented in black font with a text height of 0.1, and the five search symbols were arranged horizontally at x positions ranging from −0.4 to 0.4. Stimuli were sampled without replacement from a fixed set of uncommon glyphs.

In approximately 48% of trials, one of the two target symbols appeared among the five search symbols (“target present” trials). In the remaining trials, neither target appeared among the search symbols (“target absent” trials). The array remained visible until the participant responded. Participants pressed the right arrow key when one of the target symbols was present and the left arrow key when no target was present.

Participants completed 10 practice trials before the experimental phase. The test phase lasted up to 120 s, with a maximum of 60 items. Behavioral outcomes included accuracy, number of attempted items, and trial-level reaction time. Summary measures included overall mean reaction time and mean reaction time for correct and incorrect responses.

### 2.7 Emotional Stroop (emotional interference)

The Emotional Stroop task was used to assess cognitive control under affective interference. Each trial presented a word randomly sampled from a set of 18 items divided into three emotional categories: negative words (“addiction,” “dependence,” “failure,” “guilt,” “anxiety,” “depression”), positive words (“relaxation,” “fun,” “tranquility,” “pleasure,” “calm,” “peace”), and neutral words (“table,” “door,” “tree,” “lamp,” “window,” “sky”). Words were displayed in one of four ink colors: red, green, blue, or yellow. Participants were instructed to identify the ink color while ignoring the emotional or semantic content of the word.

The response mapping was identical to that used in the classic Stroop task: left = red, right = green, up = blue, and down = yellow. Word and ink color were selected independently, with the restriction that the same ink color could not appear on two consecutive trials. This procedure yielded approximately balanced exposure to negative, positive, and neutral stimuli, although the exact order of stimuli varied across participants due to randomization.

Each stimulus remained on screen until the participant responded, and the next trial began immediately after response registration. Participants completed 10 practice trials before the experimental phase. The test phase consisted of 100 trials. For each trial, the program recorded the word, emotional category, ink color, response accuracy, and reaction time in milliseconds.

Behavioral outcomes included overall accuracy, overall mean reaction time, and mean reaction time stratified by response accuracy and word category. The primary behavioral index of emotional interference was performance for negative emotional stimuli, given the study focus on affective distraction and cognitive-affective control.

### 2.8 Continuous performance task, CPT (sustained attention and inhibitory control)

The Continuous Performance Task was used as a non-emotional measure of sustained attention and inhibitory control. The task began with a nine-digit countdown presented in the same visual format as the subsequent task stimuli. Participants then completed 360 trials consisting of 80 target trials and 280 non-target trials. Target trials consisted of the letter “X” and represented 22.2% of trials. Non-target trials consisted of other uppercase Roman letters and represented 77.8% of trials. Stimuli were presented in random order as white characters over a dynamic white-noise background.

Each stimulus was displayed for 500 ms and was followed by a 1,000 ms inter-trial interval, resulting in a total task duration of approximately 6 min 25 s. Participants were instructed to press a keyboard key whenever the letter “X” appeared and to withhold responses to all other letters.

Behavioral outcomes included commission errors, defined as responses to non-target stimuli; omission errors, defined as missed responses to target stimuli; mean reaction time; and intra-individual reaction-time variability.

### 2.9 fNIRS recording: acquisition and preprocessing

**Figure 1:**
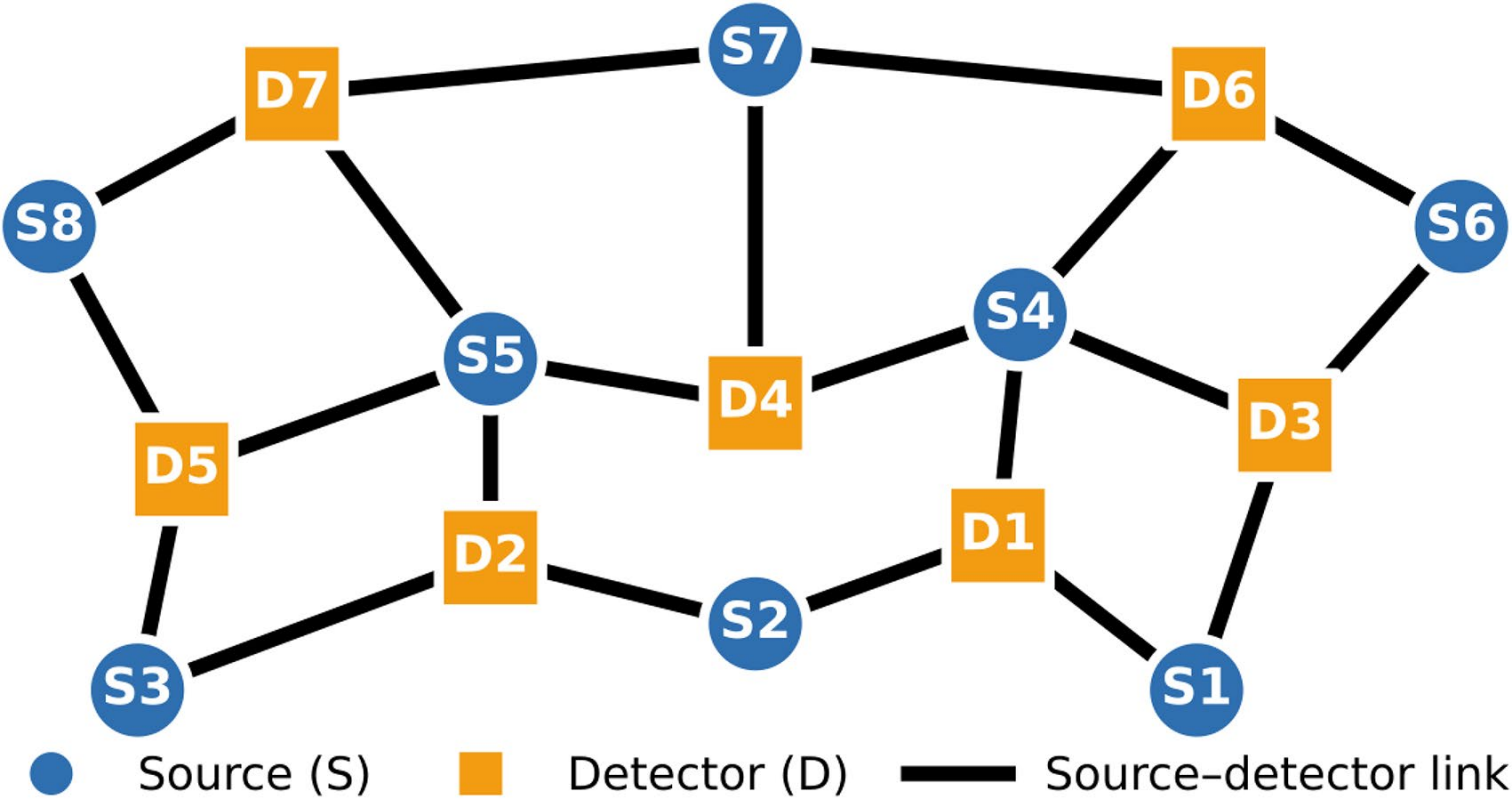
fNIRS optode montage and source–detector geometry. Blue circles denote light sources (S) and orange squares denote detectors (D). Colored line segments indicate the source–detector pairs used to define measurement channels in the prefrontal montage, as implemented in the present acquisition setup. This schematic summarizes the spatial layout underlying the channel-level and ROI-based analyses reported in the manuscript.

Hemodynamic activity was recorded using a NIRx NIRSport 2 system with a prefrontal source–detector montage optimized for bilateral prefrontal coverage. The optode layout used in the present acquisition setup is shown in Figure 1. Briefly, the montage was arranged over the prefrontal scalp and included light sources and detectors distributed bilaterally, generating source–detector measurement links across medial, lateral, and superior prefrontal sectors. This configuration was selected to capture prefrontal hemodynamic activity during tasks involving cognitive control, sustained attention, and emotional interference.

Raw light-intensity signals were converted to optical density using a logarithmic transformation. A band-pass filter of 0.01–0.20 Hz was then applied to attenuate slow drift and reduce high-frequency physiological components, including respiratory and cardiac-related fluctuations. Motion artifacts were corrected channel-wise using Temporal Derivative Distribution Repair (TDDR), a method designed to reduce abrupt motion-related signal changes in fNIRS recordings (Fishburn et al., 2019). Optical-density data were subsequently converted to oxygenated hemoglobin (HbO) and deoxygenated hemoglobin (HbR) concentration changes using the Modified Beer–Lambert Law, with differential pathlength factors set to 6/6.

The same acquisition configuration and preprocessing pipeline were applied to PRE and POST recordings to ensure comparability across time points. The resulting HbO and HbR time series were then used for event/block extraction and ROI-based analyses as described below.

### 2.10 Events and hemodynamic response windows

Task events were identified from task annotations whenever available. When annotations were incomplete or missing, task onsets and blocks were reconstructed from the experimental programming and timing structure. Blocks or events that did not contain a complete hemodynamic response window were excluded from analysis. The same event-definition procedure was applied to PRE and POST sessions.

For each task, fNIRS responses were summarized using predefined post-stimulus or post-block hemodynamic windows selected to capture the expected delayed cortical hemodynamic response. Three windows were extracted: w4_12, corresponding to 4–12 s after event or block onset; w5_15, corresponding to 5–15 s; and w6_16, corresponding to 6–16 s. The w5_15 window was treated as the primary analysis window, whereas w4_12 and w6_16 were used as sensitivity windows to assess the robustness of the results to small variations in hemodynamic timing.

No additional condition-specific baseline subtraction was applied at the extraction stage. Instead, HbO and HbR concentration-change values were obtained from the standardized preprocessed time series, and mean values were extracted from the predefined post-event/post-block windows using the same procedure for PRE and POST sessions. This approach ensured that all groups and time points were processed using identical temporal windows and extraction rules.

For each participant, values were averaged across valid events or blocks within each task and session. This yielded one summary value per participant for each combination of hemoglobin signal (HbO/HbR), ROI, task, time point, and hemodynamic window. These extracted mean concentration-change values constituted the quantitative fNIRS activation indices used in the statistical analyses.

### 2.11 Regions of interest and lateralization

The complete source–detector geometry of the prefrontal montage is shown in Figure 1 and detailed in Supplementary Table S1. The montage included eight light sources and seven detectors, yielding 21 source–detector measurement links distributed over bilateral prefrontal regions. Channel-to-region assignments were based on the spatial layout of the standard prefrontal montage and should therefore be interpreted as approximate prefrontal projections rather than individual anatomical coordinates.

Regions of interest were defined *a priori* from the prefrontal fNIRS montage. Channels were assigned to left or right prefrontal ROIs according to the source–detector midpoint and its mediolateral position relative to the midline. Channels located near the midline were assigned using a geometric criterion based on the midpoint coordinates. The bilateral prefrontal ROI was calculated as the mean of the left and right prefrontal ROIs.

The left and right ROIs were intended to capture broad lateral prefrontal activity, including approximate dorsolateral and ventrolateral prefrontal regions, whereas the bilateral ROI summarized overall prefrontal engagement across hemispheres. The anatomical interpretation of these ROIs was based on the approximate projection of the source–detector channels over prefrontal scalp regions and, where available, on 10–20/MNI-based spatial registration procedures commonly used for fNIRS channel-to-cortex approximation (Tsuzuki and Dan, 2014). Because fNIRS provides limited spatial specificity compared with MRI-based neuroimaging, the ROIs were interpreted conservatively as broad prefrontal regions rather than as precise anatomical subdivisions.

The selection of prefrontal ROIs was theoretically motivated by the role of lateral prefrontal cortex in cognitive control, inhibitory regulation, and the suppression of task-irrelevant information. These processes are central to both the classic Stroop and Emotional Stroop paradigms. In the context of cannabis use, prefrontal and fronto-limbic circuits are particularly relevant because THC and cannabis-use patterns have been associated with altered prefrontal activation and altered functional coupling during emotional processing and cognitive control. The right prefrontal ROI was of particular interest because right-lateralized prefrontal mechanisms have been implicated in inhibitory control and the regulation of emotionally salient interference.

A lateralization index was calculated to characterize hemispheric asymmetry during task performance:


LI=(L−R)/(∣L∣+∣R∣+ε)

Where L and R represent the left and right ROI values, respectively, and ε is a small constant added to avoid division by zero. Positive and negative LI values indicate relative leftward or rightward predominance, respectively. LI values were interpreted as exploratory indices of functional asymmetry rather than as primary outcome measures.

### 2.12 Quality control and inclusion criteria

A minimum of *n* ≥ 3 PRE–POST pairs per cell (hemoglobin × ROI × task × window) was required to run paired/mixed statistical tests, and *n* ≥ 3 participants per group was required for the Bayesian analysis. PRE–POST pairing was verified at the subject level, and blocks lacking a complete hemodynamic response function (HRF) window were excluded. These criteria, together with a standardized preprocessing pipeline, were intended to maximize internal validity and reproducibility of the hemodynamic metrics.

### 2.13 Behavioral analysis

Normality was assessed using Kolmogorov–Smirnov and Shapiro–Wilk tests by group and variable. Given heterogeneity of distributions (including violations in Emotional Stroop variables), a non-parametric strategy was adopted: Wilcoxon signed-rank tests for PRE vs. POST comparisons within each group; Kruskal–Wallis tests for between-group comparisons; and, when appropriate, Mann–Whitney U tests for pairwise contrasts. Tests were reported with two-tailed *p*-values and effect direction.

#### 2.13.1 Statistical considerations

The sample included participants older than 18 years, with an unequal sex distribution across groups. Given the small sample size and the absence of a balanced distribution of age and sex across groups, these variables were not included as factors or covariates in the inferential analyses to avoid unstable estimates and overfitting. Accordingly, age and sex are reported descriptively only.

### 2.14 fNIRS analysis (frequentist)

Analyses were stratified by hemoglobin (HbO/HbR) and by task × ROI, controlling multiplicity using false discovery rate (FDR; Benjamini–Hochberg, *q* = 0.05) within families defined by hemoglobin and test type (e.g., all likelihood-ratio tests for the time×group interaction in HbR). The following were estimated:

- Within-group change on *Δ* = POST − PRE (paired *t*-test; *t*, *p*, d_z, 95% CI; FDR correction by hemoglobin);
- Between-group comparisons at PRE and POST (Welch’s *t*-test; *t*, *p*, Hedges’ g, 95% CI; FDR correction by hemoglobin and time point);
- A confirmatory linear mixed-effects model for each hemoglobin × ROI × task combination [value ~ time × group + (1|subject)], using likelihood-ratio tests (LRT) for time, group, and the time × group interaction, and reporting the POST coefficient (βpost) from the full model;
- An ANCOVA on POST adjusted for PRE (POST ~ PRE + Group; OLS Type II), reporting F, p, FDR-adjusted *p* values, partial η^2^, and between-group contrasts with FDR-adjusted p values; Hedges’ g was computed on residualized POST values.

The w5_15 window was prioritized as the primary window, with w4_12 and w6_16 treated as sensitivity analyses.

### 2.15 fNIRS analysis (Bayesian)

For each task × window (w4_12, w5_15, w6_16) × metric (l_hbo, r_hbo, bilat_hbo, li_hbo, l_hbr, r_hbr, bilat_hbr, li_hbr), *Δ* = POST − PRE was modeled by group as follows:


Δi~Normal(μG,σG),μG~Normal(0,1),σG~HalfNormal (1)

Inference was performed in PyMC using NUTS (draws = 1,000; tune = 1,000; chains = 2; target_accept = 0.9), summarizing posterior means and 95% HDIs, and monitoring R-hat (R-hat) and effective sample size (ESS). Between-group contrasts were computed on μA − μB [mean, 95% HDI, P(diff > 0)]. Criterion: a 95% HDI excluding 0 was interpreted as consistent evidence; if 0 was included, results were considered a trend/non-conclusive.

### 2.16 Software and reproducibility

The processing and analysis pipeline was implemented in Python (3.x) using MNE-Python and MNE-NIRS for .snirf reading, annotation synchronization, and epoch construction; optical density conversion, 0.01–0.20 Hz band-pass filtering, TDDR motion correction, and MBLL (DPF = 6/6) to derive HbO/HbR signals; pandas/numpy for data reshaping and aggregation; scipy/statsmodels for frequentist analyses; PyMC/ArviZ for Bayesian modeling; and matplotlib for visualization. Batch automation and export were performed via dedicated workflow scripts (e.g., *fnirs_batch_export_all_windows_blocks.py*, *bayes_prepost_groups_alltasks.py*), which generate the CSV files used in the report, thereby supporting reproducibility.

## 3 Results

### 3.1 Behavioral performance

Behavioral findings were concentrated in the Emotional Stroop task. Within-group PRE–POST comparisons showed that occasional cannabis users exhibited a significant reduction in negative-stimulus reaction time from PRE to POST (*Z* = −2.547, *p* = 0.011). In contrast, the PRE–POST change in the number of correct responses to negative stimuli did not reach statistical significance in occasional users (*Z* = −1.511, *p* = 0.131). This pattern indicates faster responses to negative emotional stimuli after the abstinence interval, without a statistically significant within-group accuracy change.

In the non-consumer group, a significant PRE–POST change was also observed for negative-stimulus reaction time (*Z* = −2.240, *p* = 0.025), suggesting a possible retest-related effect for negative emotional stimuli. The PRE–POST change in correct responses was not significant in this group (*Z* = −0.816, *p* = 0.414). Frequent/chronic cannabis users did not show significant PRE–POST behavioral changes in either negative-stimulus reaction time (*Z* = −1.599, *p* = 0.110) or correct responses (*Z* = −0.272, *p* = 0.785).

Between-group analyses further supported the selective involvement of Emotional Stroop performance. Kruskal–Wallis tests showed significant group differences for negative-stimulus reaction time (*H* = 6.42, *p* = 0.040) and number of correct responses to negative stimuli (*H* = 6.56, *p* = 0.038). Pairwise Mann–Whitney U comparisons indicated no significant POST difference between non-consumers and occasional users in negative-stimulus reaction time (U = 24, *p* = 0.248). For correct responses, the non-consumer versus occasional-user comparison showed U = 16, with an asymptotic *p* value of 0.041 and a trend-level exact p value of 0.059. Occasional users differed significantly from frequent/chronic users in both negative-stimulus reaction time (U = 14, *p* = 0.019) and correct responses (U = 16.5, *p* = 0.024). No significant differences were observed between non-consumers and frequent/chronic users in negative-stimulus reaction time (U = 19, *p* = 0.102) or correct responses (U = 34.5, *p* = 0.865).

**Figure 2:**
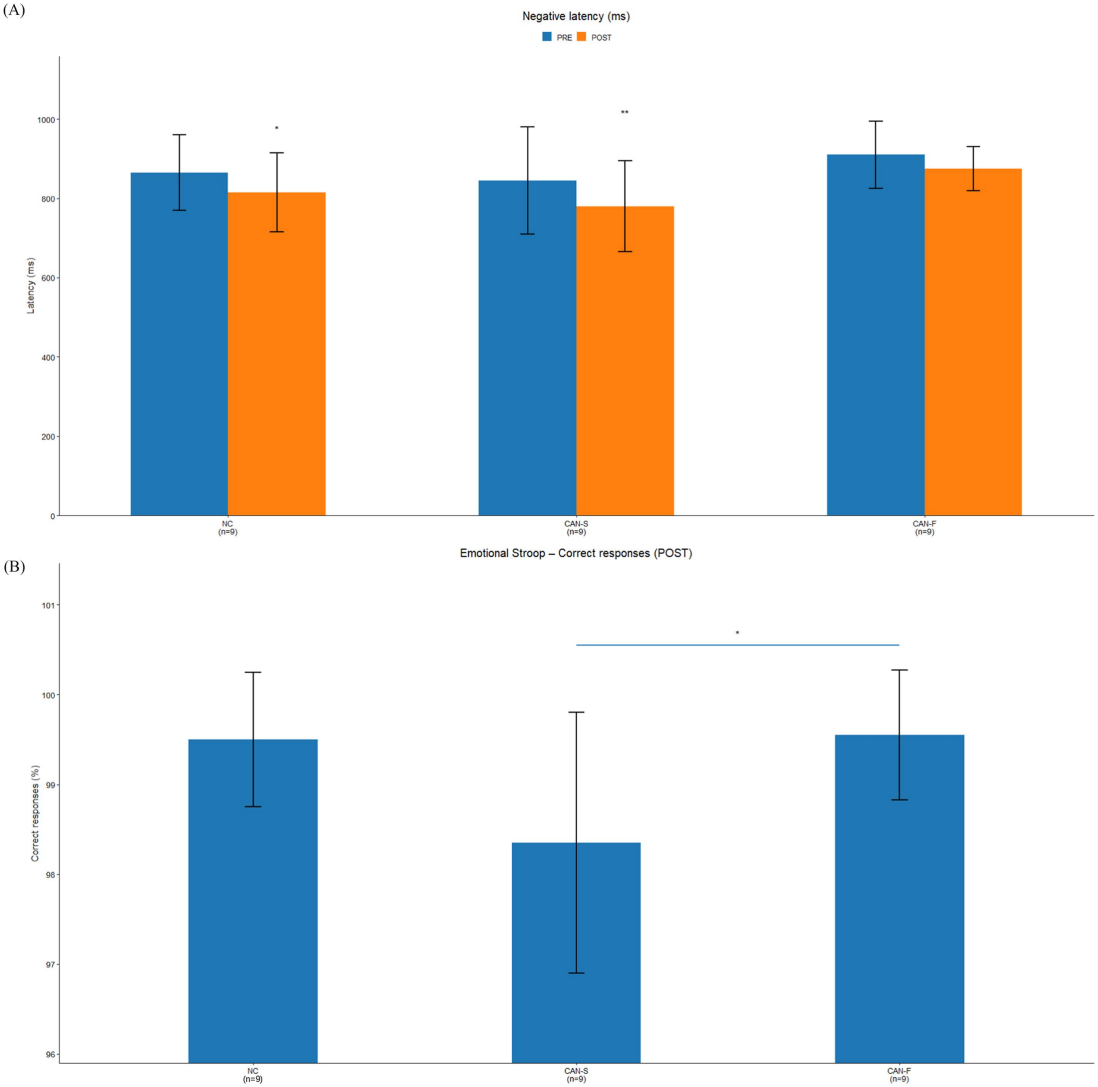
Emotional Stroop performance. **(A)** Negative latency (ms) for the Control, Occasional (sporadic), and Chronic groups at PRE and POST. Bars show group means and error bars indicate standard deviations (SD). Asterisks denote within-group PRE POST differences (Wilcoxon signed-rank test): *p* ≤ 0.05 (*) and *p* ≤ 0.01 (**). **(B)** Correct responses (POST) across groups. Bars show POST means with SD. The horizontal line and asterisk indicate a significant between-group difference at POST between Occasional and Chronic users (Mann–Whitney U test; two-tailed *p* ≤ 0.05). Sample sizes are shown under each bar.

By contrast, no significant behavioral effects were observed for the non-emotional tasks. Specifically, CPT, Symbol Search, and classic Stroop outcomes did not show significant between-group differences or within-group PRE–POST changes under the planned non-parametric approach. Overall, the behavioral results indicate that the main behavioral effects were selective to negative emotional interference in the Emotional Stroop task, rather than reflecting a generalized alteration across the full cognitive battery (see Figure 2; Table 1).

**Table 1:** Nonparametric tests for emotional stroop outcomes.

Outcome	Comparison	Test	Statistic	*p*
Negative latency (ms)	Controls: PRE vs. POST	Wilcoxon	**Z = −2.240**	**0.025**
Negative latency (ms)	Occasional: PRE vs. POST	Wilcoxon	**Z = −2.547**	**0.011**
Negative latency (ms)	Chronic: PRE vs. POST	Wilcoxon	Z = −1.599	0.110
Correct responses (count)	Controls: PRE vs. POST	Wilcoxon	Z = −0.816	0.414
Correct responses (count)	Occasional: PRE vs. POST	Wilcoxon	Z = −1.511	0.131
Correct responses (count)	Chronic: PRE vs. POST	Wilcoxon	Z = −0.272	0.785
Negative latency (ms)	POST: Controls vs. Occasional	Mann–Whitney	U = 24	0.248
Negative latency (ms)	POST: Occasional vs. Chronic	Mann–Whitney	**U = 14**	**0.019**
Negative latency (ms)	POST: Controls vs. Chronic	Mann–Whitney	U = 19	0.102
Correct responses (count)	POST: Controls vs. Occasional	Mann–Whitney	U = 16	0.041 (asympt.) / 0.059* (exact)
Correct responses (count)	POST: Occasional vs. Chronic	Mann–Whitney	**U = 16.5**	**0.024**
Correct responses (count)	POST: Controls vs. Chronic	Mann–Whitney	U = 34.5	0.865

Two-tailed *p* values are reported. “ns” denotes non-significant results (*p* > 0.05). Kruskal–Wallis and Mann–Whitney tests were performed on POST variables (as labeled in the SPSS output). *Trend-level exact *p* reported in the SPSS output (Control vs Occasional for correct responses). Wilcoxon Z and *p* values were taken from the provided SPSS output tables for PRE–POST comparisons. Bold values indicate statistically significant results (*p* < 0.05).

### 3.2 fNIRS findings

#### 3.2.1 Quantitative activation indices and analysis framework

The quantitative fNIRS activation indices were the mean HbO and HbR concentration-change values extracted from the predefined hemodynamic response windows for each participant, task, ROI, time point, and hemoglobin signal. The w5_15 window was treated as the primary analysis window, whereas w4_12 and w6_16 were used as sensitivity windows. For the mixed-effects models, these extracted concentration-change values were entered as the dependent variable, and the main inferential focus was the time × group interaction. Model estimates are reported as fixed-effect coefficients, and *p*-values were adjusted using FDR correction within the predefined statistical families.

#### 3.2.2 Primary mixed-effects results

The main FDR-corrected fNIRS effect was observed for HbR during the Emotional Stroop task. The linear mixed-effects model showed a significant time × group interaction in the right prefrontal ROI (FDR-adjusted *p* ≈ 3 × 10^−4^; βpost ≈ − 3.64 × 10^−8^), with a parallel significant effect in the bilateral prefrontal ROI (FDR-adjusted *p* ≈ 3 × 10^−4^; βpost ≈ − 2.94 × 10^−8^). This pattern indicates that pre–post HbR modulation during emotional interference differed across groups, with the clearest contrast involving non-consumers and occasional cannabis users.

#### 3.2.3 Non-significant corrected analyses

No FDR-corrected significant within-group PRE–POST effects were observed when ROI × task × hemoglobin combinations were analyzed separately. Similarly, the POST ANCOVA adjusted for PRE values did not show FDR-corrected significant global group effects (FDR-adjusted *p* ≥ 0.56; η^2^p ≤ ~0.14). Therefore, these analyses are interpreted as non-significant in the corrected frequentist framework.

For the non-emotional tasks, including CPT and Symbol Search, no robust FDR-corrected fNIRS effects were observed in the frequentist analyses. This indicates that the corrected hemodynamic findings were not generalized across the full cognitive battery, but were concentrated in the Emotional Stroop task.

#### 3.2.4 Bayesian contrasts and exploratory convergence

**Figure 3:**
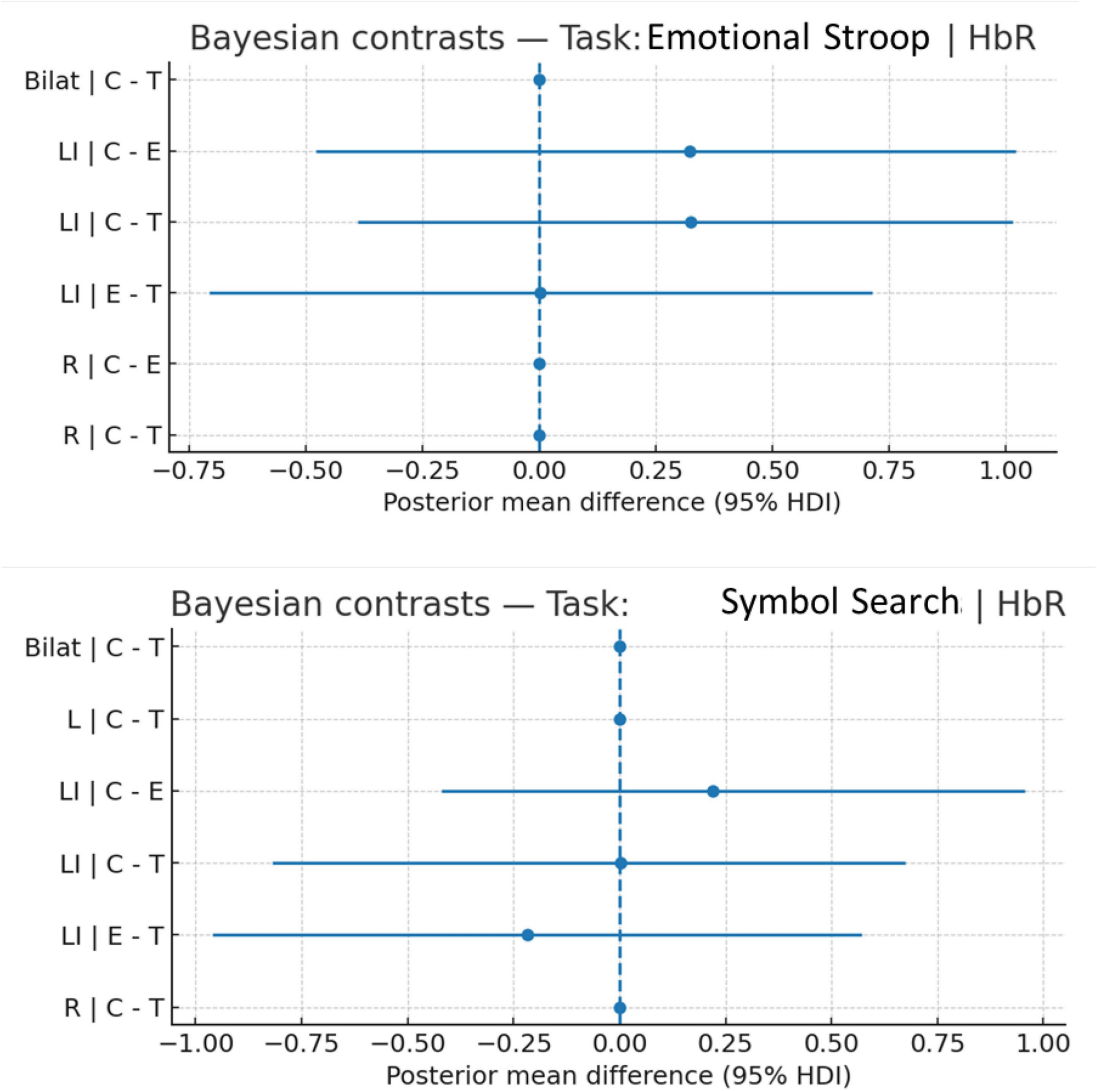
Bayesian contrasts for ΔHbR (POST − PRE) in Emotional Stroop (top) and Symbol Search (bottom). Panels show posterior mean differences (μ_A − μ_B) with 95% highest density intervals (HDI) for prefrontal ROIs (Bilateral; Laterality Index [LI]; and Left/Right as shown). The vertical dashed line marks 0 (no difference). Points indicate posterior means and horizontal segments indicate 95% HDIs. Groups: C = controls; E = occasional users; T = chronic/regular users. Positive values indicate μ_A > μ_B.

The Bayesian analysis of POST–PRE change scores provided convergent evidence for the right prefrontal HbR effect during the Emotional Stroop task. The Control − Occasional contrast showed a 95% HDI excluding zero in the right ROI (mean ≈ − 4.06 × 10^−8^; 95% HDI [−7.62 × 10^−8^, −0.82 × 10^−8^]; P(diff > 0) ≈ 0.0095). Bayesian evidence for HbO and lateralization-index measures was weak or inconclusive. Although the Bayesian framework suggested a possible Control − Chronic difference in Symbol Search for bilateral HbR, this pattern was not confirmed by the frequentist analysis and is therefore treated as exploratory and non-conclusive (see Figure 3).

**Figure 4:**
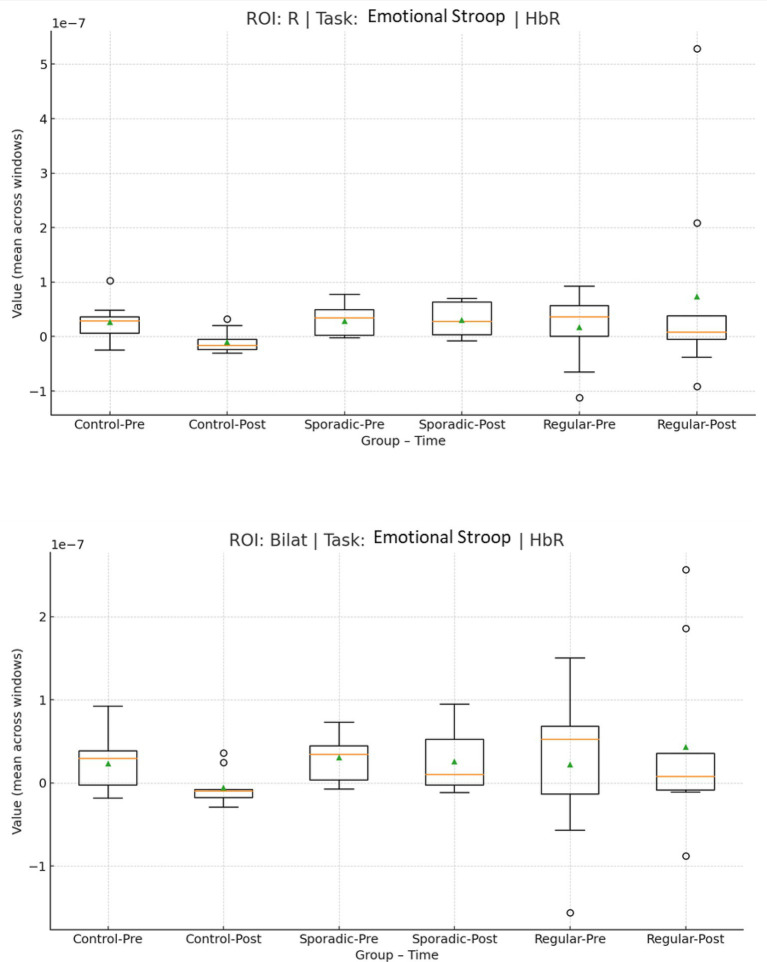
Group-by-time distributions—Emotional Stroop, HbR (Right and Bilateral ROIs). Boxplots show HbR values (averaged across HRF response windows) at PRE and POST for each group (Control, Sporadic, Regular/Chronic) in the right (R; top panel) and bilateral (Bilat; bottom panel) prefrontal ROIs. Boxes represent the interquartile range (IQR), the central line indicates the median, whiskers extend to 1.5 × IQR, green triangles denote the mean, and open circles indicate outliers. The y-axis is expressed in units of 10^−7^.

Exploratory uncorrected POST comparisons were directionally consistent with the mixed-effects model for Emotional Stroop–HbR, particularly for non-consumers versus occasional users in the right prefrontal ROI (*p* ≈ 0.0096; Hedges’ *g* ≈ −1.35) and bilateral ROI (*p* ≈ 0.031; Hedges’ *g* ≈ −1.18). Because these contrasts were not the primary corrected tests, they are reported only as exploratory convergence with the corrected mixed-effects finding. Descriptively, the PRE–POST distributions of HbR values in the right and bilateral ROIs also illustrate this group-dependent modulation during Emotional Stroop performance (see Figure 4).

#### 3.2.5 Channel-level visualizations

**Figure 5:**
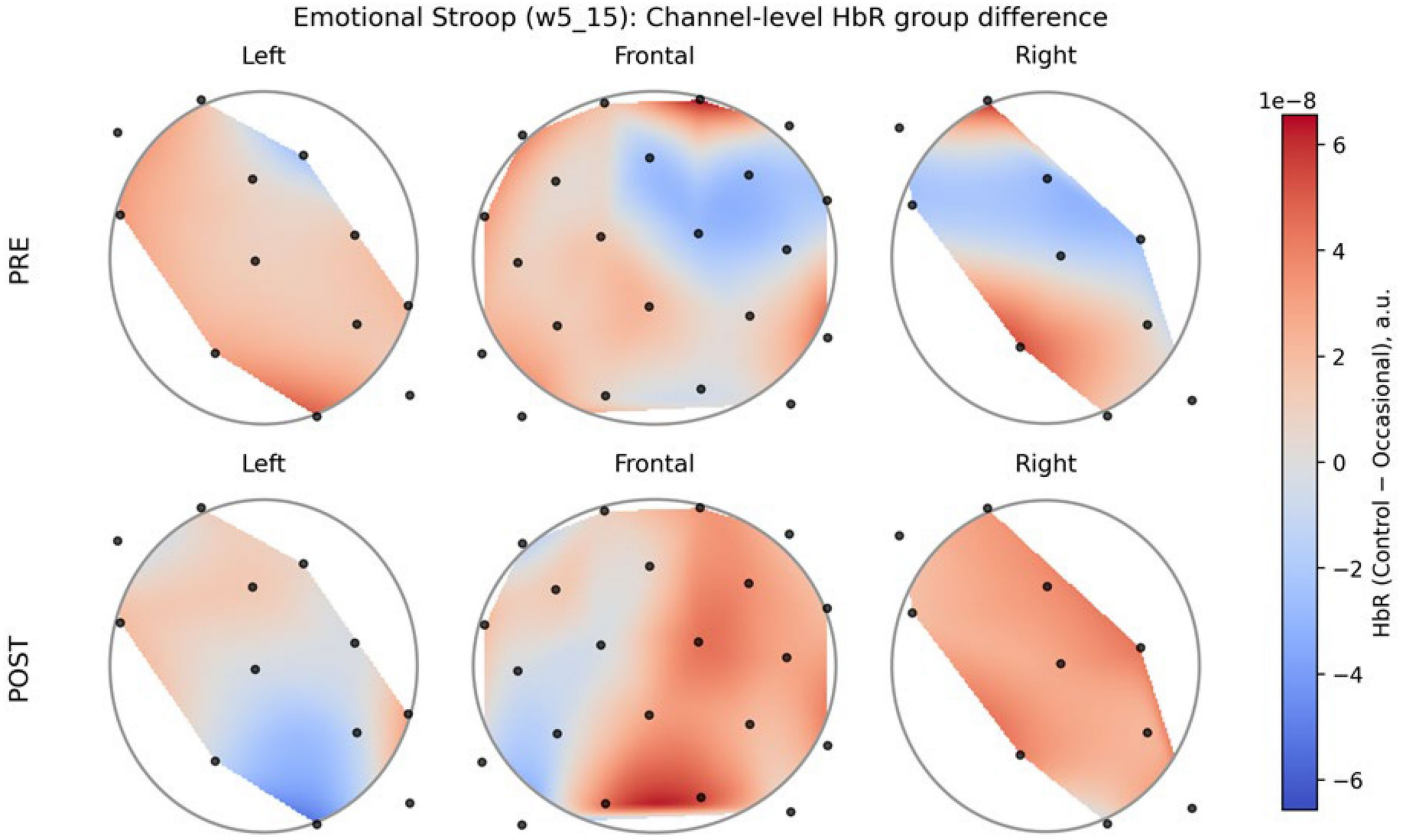
Emotional Stroop (w5_15): channel-level HbR group contrast (Control − Occasional) at PRE and POST. The top panels depict the pre-intervention (PRE) group contrast and the bottom panels depict the post-intervention (POST) contrast, shown in left, frontal, and right views of the prefrontal montage. Color values represent HbR(Control) − HbR(Occasional) (a.u.) with the scale centered at 0; warmer colors indicate relatively higher HbR in controls, whereas cooler colors indicate relatively higher HbR in occasional users. The maps indicate a change in the between-group topography from PRE to POST, with the clearest separation in the right prefrontal view at POST, consistent with the observed time × group modulation in HbR during the Emotional Stroop task. Black dots indicate channel locations; surfaces are interpolated for visualization.

**Figure 6:**
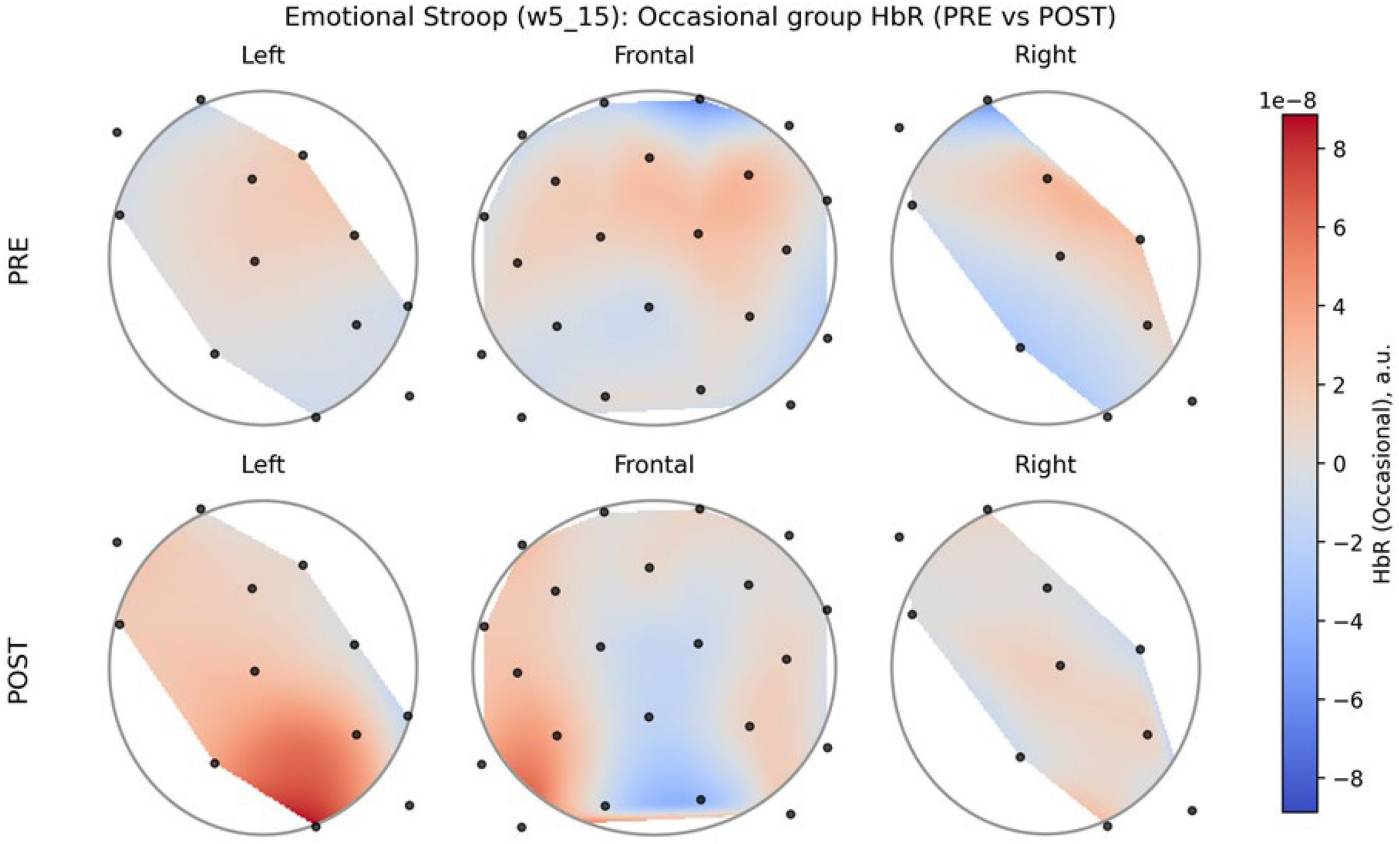
Emotional Stroop (w5_15): Occasional-use group HbR (PRE vs. POST). Compared with PRE (top row), POST (bottom row) shows a more pronounced HbR decrease over the right prefrontal region (right column; more negative values/greater task-related deoxygenation), with a weaker but spatially similar pattern in the frontal/bilateral view. This visual shift is consistent with the main statistical finding of a time × group effect in HbR during the Emotional Stroop that was driven by differential PRE–POST modulation in the right (and bilateral) ROI, indicating altered prefrontal hemodynamic engagement after the ≥72-h abstinence interval in occasional users. Black dots mark channel locations; color maps are interpolated for visualization (scale centered at 0; a.u.).

The channel-level visualizations provided complementary descriptive information on the spatial distribution of the Emotional Stroop HbR effect. The Control − Occasional channel-level contrast showed a change in between-group topography from PRE to POST, with the clearest separation in the right prefrontal view at POST. In addition, the PRE–POST visualization within the occasional-use group suggested a more pronounced HbR decrease over the right prefrontal region after the abstinence interval. These topographic maps are presented as descriptive visualizations of the ROI-level findings and should not be interpreted as independent channel-wise inferential tests (see Figures 5, 6).

#### 3.2.6 Overall fNIRS pattern

Overall, the fNIRS findings indicate a selective right and bilateral prefrontal HbR modulation during Emotional Stroop performance, rather than a generalized hemodynamic alteration across all cognitive tasks or hemoglobin signals. Together with the behavioral findings, this pattern suggests that the core results of the study were concentrated on negative emotional interference in the Emotional Stroop task, with occasional cannabis users showing faster responses to negative emotional stimuli, an accuracy-related between-group pattern, and convergent right-prefrontal HbR modulation. Detailed ROI-wise, channel-wise, and sensitivity-window results are provided in the Supplementary materials to improve readability of the main text.

## 4 Discussion

This pilot pre–post study examined whether short-term abstinence-related differences across cannabis-use profiles were preferentially expressed during emotional interference and whether these differences were accompanied by prefrontal hemodynamic modulation measured with fNIRS. The main findings were concentrated in the Emotional Stroop task rather than across the full cognitive battery. Occasional cannabis users showed the clearest behavioral shift after the abstinence interval, characterized primarily by faster responses to negative emotional stimuli, together with an accuracy-related pattern observed in the between-group comparisons. In parallel, fNIRS analyses showed a time × group interaction for HbR in right and bilateral prefrontal ROIs during the Emotional Stroop task. By contrast, chronic users did not show reliable behavioral alteration in this task battery, and non-emotional tasks, including CPT, Symbol Search, and the classic Stroop, did not show robust behavioral or fNIRS effects. Taken together, these findings suggest, in a preliminary and hypothesis-generating manner, that emotional interference may be more sensitive than non-emotional cognitive performance for detecting short-term, use-pattern-dependent differences in adult cannabis users.

The behavioral pattern observed in occasional users was mainly driven by reduced response latency for negative emotional stimuli. Rather than indicating a generalized cognitive deficit, the results point to a more specific alteration in performance when emotionally salient negative material must be ignored. This is relevant because the Emotional Stroop requires participants to suppress the influence of affective word content while maintaining goal-directed responses to non-emotional stimulus features. Although the accuracy findings did not show a significant within-group PRE–POST change in occasional users, the between-group pattern for correct responses suggests that faster responding may have occurred in the context of a possible accuracy-related cost. This pattern is therefore better interpreted as suggestive of a possible speed–accuracy trade-off, rather than as definitive evidence of one. The absence of comparable effects in CPT, Symbol Search, and classic Stroop suggests that the observed pattern was not simply attributable to generalized slowing, broad attentional impairment, or nonspecific task difficulty. Instead, the findings support the possibility that cannabis-related differences may become more evident when cognitive control is challenged by emotional distraction. However, the control group also showed a PRE–POST change in negative-stimulus latency, indicating that retest, habituation, sensitization, fatigue, or state-dependent factors may influence repeated Emotional Stroop performance. Previous work has shown that repeated exposure and emotional arousal can modulate Emotional Stroop interference, particularly for negative material, and that state anxiety may influence interference effects (Ben-Haim et al., 2014; Dresler et al., 2009). Therefore, the behavioral findings should be interpreted cautiously and require replication using larger samples, counterbalanced designs, and alternate stimulus sets.

One possible interpretation is that intermittent cannabis exposure may be associated with greater short-term vulnerability of cognitive-affective control than more frequent use, at least within the brief abstinence window examined here. This interpretation is broadly compatible with evidence suggesting that acute THC-related cognitive disruption may be more pronounced in occasional users than in frequent users, consistent with tolerance-like functional adaptation (Colizzi and Bhattacharyya, 2018; Ramaekers et al., 2016). Naturalistic pre–post evidence has also reported more marked acute slowing and short-term memory decrements in occasional users compared with daily users (Brooks-Russell et al., 2024). Nevertheless, the present data cannot establish tolerance as a mechanism. The apparently preserved performance of chronic users could reflect reduced acute sensitivity, different recovery kinetics after abstinence, self-selection of individuals more resilient to cannabis-related cognitive effects, or insufficient sensitivity of the task battery to detect chronic-use-related alterations. Prior reviews emphasize that cannabis-related cognitive outcomes vary according to cumulative exposure, abstinence duration, developmental timing, and individual vulnerability (Crane et al., 2013; Kroon et al., 2020; Wade et al., 2024). Thus, the absence of robust behavioral effects in chronic users should not be interpreted as evidence of absence of harm.

The fNIRS findings provide preliminary neurofunctional support for the behavioral pattern observed during emotional interference. The main corrected hemodynamic result was a time × group interaction for HbR in right and bilateral prefrontal ROIs during the Emotional Stroop task, with convergent exploratory evidence from Bayesian contrasts and descriptive channel-level visualizations. Right-lateralized prefrontal involvement is broadly consistent with the role of lateral prefrontal networks in inhibitory control, emotional regulation, and the suppression of task-irrelevant affective information. Previous fNIRS studies using Stroop-like paradigms have reported altered prefrontal engagement in conditions characterized by heightened interference from negative material, including PTSD and depression (Yennu et al., 2016; Verma et al., 2024). In the present study, the combination of faster responses, an accuracy-related between-group pattern, and altered right-prefrontal HbR modulation in occasional users may reflect less efficient cognitive-affective control under emotional distraction. However, because individual brain–behavior coupling was not directly tested, and because the accuracy-related evidence was not based on a significant within-group PRE–POST accuracy change, this interpretation remains provisional.

The HbR nature of the main fNIRS finding also requires caution. Although HbR can provide meaningful information about task-related hemodynamic responses, HbO is often considered the more sensitive and consistently observed fNIRS marker of cortical activation. HbR changes may be smaller, more variable, and physiologically complex, especially when capillary blood flow, oxygen extraction, vascular volume, and systemic factors change simultaneously. Therefore, the present HbR findings should be viewed as preliminary evidence of altered prefrontal hemodynamic modulation rather than definitive evidence of a specific neural mechanism. This caution is particularly important because the study did not include short-separation channels and was restricted to prefrontal coverage. Accordingly, replication in larger samples with improved physiological control, broader cortical coverage, and direct brain–behavior modeling is necessary before firm mechanistic conclusions can be drawn.

The present results also fit within a broader neuroimaging literature suggesting that cannabis use may affect prefrontal and fronto-limbic systems involved in cognitive control, emotion regulation, and reward-related processing. Functional neuroimaging studies have reported altered medial and lateral prefrontal activity during emotional evaluation and emotion regulation among regular cannabis users (Wesley et al., 2016; Zimmermann et al., 2017). Other work suggests that some cannabis-related alterations may be state-dependent and may partially normalize after longer abstinence periods (Zimmermann et al., 2018). Meta-analytic neuroimaging evidence also points to convergent alterations in brain regions supporting cognitive control and reward processing among cannabis users (Yanes et al., 2018). The present fNIRS findings complement this literature by suggesting that prefrontal hemodynamic modulation during emotional interference may help characterize cannabis-related functional variability across different use profiles. However, the small sample size, self-reported abstinence, and absence of detailed cumulative exposure measures prevent strong conclusions regarding chronicity, causality, or clinical significance.

The Emotional Stroop findings may also be considered within the broader context of addiction-related prefrontal dysfunction. Substance use disorders are often associated with alterations in inhibitory control, salience processing, emotion regulation, and relapse vulnerability, processes that depend partly on prefrontal and fronto-limbic circuits. Emotionally salient cues or negative affective states can increase cognitive load and interfere with goal-directed control, potentially making affect-laden tasks more sensitive than purely non-emotional paradigms. In this sense, the selective pattern observed here suggests that negative emotional interference may provide a useful experimental context for probing subtle cannabis-related differences in cognitive-affective regulation. This interpretation is consistent with the view that cannabis-related neurocognitive effects are often subtle, domain-specific, and moderated by use history rather than uniformly generalized across tasks (Cuttler et al., 2023; Dellazizzo et al., 2022; Kroon et al., 2020). At the same time, the findings should not be generalized to clinical cannabis use disorder or relapse risk without further validation.

The absence of robust effects in the non-emotional tasks is also informative. CPT, Symbol Search, and classic Stroop did not show significant behavioral differences or robust FDR-corrected fNIRS effects. This negative pattern suggests that the present effects were not generalized across sustained attention, visual search, processing speed, or non-emotional cognitive interference. Prior studies have shown that residual or abstinence-related cognitive effects may vary substantially according to domain, developmental stage, cannabis potency, cumulative exposure, and abstinence duration (Crane et al., 2013; Hanson et al., 2010; Goud et al., 2022; Lorenzetti et al., 2016). Therefore, the absence of effects in these tasks should be interpreted as task- and sample-specific rather than as evidence that cannabis use has no impact on non-emotional cognition. Larger studies may be able to determine whether emotional interference is genuinely more sensitive than non-emotional performance or whether the present pattern reflects the limited power and specific characteristics of this pilot sample.

From an applied perspective, the findings should be translated with considerable caution. If replicated, the pattern observed in occasional users could be relevant to everyday situations that require efficient attention, emotional filtering, and rapid decision-making under distraction. Evidence from psychomotor and driving-related studies suggests that cannabis-related impairment may vary according to use history and may be more evident in less tolerant or occasional users (Ramaekers et al., 2009; McCartney et al., 2022; Asbridge et al., 2012; Simmons et al., 2022). However, the present study did not assess driving, operational performance, clinical outcomes, or real-world risk directly. Therefore, any practical implications should be considered exploratory. Similarly, the relatively preserved performance observed in chronic users within this brief task battery should not be interpreted as evidence of safety, because functional tolerance may coexist with neurobiological adaptation and longer-term cognitive or affective risks (Lorenzetti et al., 2016; Kroon et al., 2020).

Methodologically, the study supports the potential value of combining affect-laden cognitive paradigms with portable neurofunctional recording. fNIRS is increasingly used as a practical tool for assessing prefrontal function in cognitive and clinical contexts (Gao et al., 2024), and previous cannabis-related fNIRS work has suggested that prefrontal hemodynamic measures may be sensitive to THC intoxication and impairment-related states (Gilman et al., 2022). The present results extend this approach by applying fNIRS to an Emotional Stroop paradigm in a pre–post abstinence design and by contrasting occasional and chronic users. Nevertheless, the current findings remain preliminary. Future studies should include larger and better-balanced samples, objective biological verification of abstinence, detailed assessment of cannabis exposure and product potency, standardized withdrawal measures, assessment of alcohol and nicotine use, short-separation fNIRS channels, and direct modeling of individual brain–behavior relationships.

In summary, this pilot study suggests that emotional interference may be a particularly informative context for detecting short-term cannabis-use-related differences in cognitive-affective control. The strongest pattern was observed in occasional users, who showed faster responses to negative Emotional Stroop stimuli, an accuracy-related between-group pattern, and convergent right-prefrontal HbR modulation after the abstinence interval. In contrast, non-emotional tasks did not show robust behavioral or hemodynamic effects, and chronic users did not show clear impairment within the present task battery. These findings should be interpreted as preliminary and hypothesis-generating. They support further investigation of emotional interference and prefrontal hemodynamic responses as potential markers of use-pattern-dependent cannabis-related functional variability, but they do not support broad claims regarding generalized cognitive impairment, tolerance, safety, or clinical risk without replication.

## 5 Limitations

This study should be interpreted explicitly as a pilot proof-of-concept investigation. First, the small sample size limits statistical power, increases the risk of false-negative findings, and may yield unstable effect estimates, particularly for chronic-user effects. The findings should therefore be considered preliminary and hypothesis-generating rather than confirmatory.

Second, abstinence was based on self-report rather than biological verification. In the absence of objective measures such as THC metabolites, residual THC exposure, withdrawal-related symptoms, or other state-dependent factors could not be quantified. Third, the cannabis-use groups were defined primarily according to frequency of use, and more detailed indices of exposure, including age of onset, years of use, estimated THC potency, cumulative exposure, and abstinence-related symptoms, were not available in sufficient detail for inferential modeling. This may have contributed to within-group heterogeneity.

Fourth, the fNIRS setup was restricted to prefrontal coverage and did not include short-separation channels. Therefore, the possibility of superficial or systemic physiological contamination cannot be excluded. Although preprocessing was standardized and included band-pass filtering and motion correction, fNIRS signals may still be influenced by scalp blood flow, autonomic changes, respiration, blood pressure variation, and other extracerebral components.

Fifth, the main hemodynamic finding involved HbR rather than HbO. This point requires particular caution. In many fNIRS studies, HbO is often considered the more sensitive and consistently observed marker of task-related activation because increases in neural activity are typically accompanied by increases in regional cerebral blood flow and oxygen delivery. HbR changes, by contrast, may be smaller, more variable, and physiologically more complex, because oxygen extraction, capillary blood flow, and vascular volume can change simultaneously. As a result, HbR does not necessarily show a simple inverse relationship with HbO. Although HbR may provide useful complementary information, HbR-based findings should be interpreted cautiously, particularly in small samples and in the absence of short-separation correction. Accordingly, the right-prefrontal HbR effect observed here should be regarded as preliminary and in need of replication.

Sixth, ROI averaging may have obscured more spatially focal channel-level effects. Conversely, channel-level analyses in a sample of this size would substantially increase the multiple-comparison burden and the risk of unstable estimates. The ROI approach was therefore chosen as a conservative strategy appropriate for a pilot study, but future research should combine larger samples with more spatially detailed modeling.

Additional limitations include retest and practice effects inherent to pre–post designs, as suggested by changes observed in controls; the absence of direct brain–behavior coupling analyses linking individual HbR modulation to individual performance change; and the lack of counterbalanced or alternate Emotional Stroop stimulus sets. Taken together, the present findings warrant replication in larger, biologically verified longitudinal samples with broader fNIRS coverage, short-separation channels, more detailed characterization of cannabis exposure, and direct modeling of brain–behavior relationships.

## 6 Conclusion

This pilot pre–post fNIRS study suggests that emotional interference may be a particularly informative context for detecting short-term, use-pattern-dependent differences in adult cannabis users. Behavioral effects were concentrated in the Emotional Stroop task, where occasional users showed faster responses to negative emotional stimuli and an accuracy-related between-group pattern after the abstinence interval. This behavioral profile was accompanied by convergent right and bilateral prefrontal HbR modulation, whereas non-emotional tasks did not show robust behavioral or hemodynamic effects.

Taken together, these preliminary findings support further investigation of affect-laden cognitive control and prefrontal hemodynamic responses as potential markers of cannabis-use-related functional variability. However, the results should be interpreted as hypothesis-generating and require replication in larger, better-characterized samples before firm mechanistic or clinical conclusions can be drawn.

## Data Availability

The raw data supporting the conclusions of this article will be made available by the authors, without undue reservation. The code used for data processing and analysis is available from the corresponding author upon reasonable request.
