# Supplementary material for: Emotional interference and right prefrontal hemodynamic responses in adult cannabis users: a pilot fNIRS study
Source: Front Psychol. 2026 Jul 24;17:1873912. doi: 10.3389/fpsyg.2026.1873912 (PMC13447055; doi:10.3389/fpsyg.2026.1873912)
Supplement: Supplementary file 1 [file Table_1.docx]

**Supplementary Table S1. fNIRS source–detector links, approximate prefrontal coverage, and ROI assignment**

| Working channel ID | Source–detector pair | Montage sector | Approximate prefrontal coverage | ROI assignment for analysis | Anatomical interpretation |
| --- | --- | --- | --- | --- | --- |
| Ch1 | S1–D1 | Right inferior/medial | Right inferior/frontopolar prefrontal region | Right prefrontal ROI | Approximate right ventral/frontopolar prefrontal channel |
| Ch2 | S1–D3 | Right lateral | Right lateral prefrontal region | Right prefrontal ROI | Approximate right lateral prefrontal channel |
| Ch3 | S2–D1 | Lower right-medial | Right medial/frontopolar prefrontal region | Right prefrontal ROI | Approximate right medial-frontopolar channel |
| Ch4 | S2–D2 | Lower left-medial | Left medial/frontopolar prefrontal region | Left prefrontal ROI | Approximate left medial-frontopolar channel |
| Ch5 | S3–D2 | Left inferior/medial | Left inferior/frontopolar prefrontal region | Left prefrontal ROI | Approximate left ventral/frontopolar prefrontal channel |
| Ch6 | S3–D5 | Left lateral | Left lateral prefrontal region | Left prefrontal ROI | Approximate left lateral prefrontal channel |
| Ch7 | S4–D1 | Right medial | Right medial/lateral prefrontal region | Right prefrontal ROI | Approximate right prefrontal channel |
| Ch8 | S4–D3 | Right lateral | Right lateral prefrontal region | Right prefrontal ROI | Approximate right lateral prefrontal channel |
| Ch9 | S4–D4 | Right centro-medial | Right medial/frontopolar prefrontal region | Right prefrontal ROI | Approximate right medial prefrontal channel |
| Ch10 | S4–D6 | Right superior/lateral | Right superior lateral prefrontal region | Right prefrontal ROI | Approximate right dorsolateral/superior prefrontal channel |
| Ch11 | S5–D2 | Left medial | Left medial/lateral prefrontal region | Left prefrontal ROI | Approximate left prefrontal channel |
| Ch12 | S5–D4 | Left centro-medial | Left medial/frontopolar prefrontal region | Left prefrontal ROI | Approximate left medial prefrontal channel |
| Ch13 | S5–D5 | Left lateral | Left lateral prefrontal region | Left prefrontal ROI | Approximate left lateral prefrontal channel |
| Ch14 | S5–D7 | Left superior/lateral | Left superior lateral prefrontal region | Left prefrontal ROI | Approximate left dorsolateral/superior prefrontal channel |
| Ch15 | S6–D3 | Right lateral | Right lateral prefrontal region | Right prefrontal ROI | Approximate right lateral prefrontal channel |
| Ch16 | S6–D6 | Right superior/lateral | Right superior lateral prefrontal region | Right prefrontal ROI | Approximate right superior prefrontal channel |
| Ch17 | S7–D4 | Midline superior | Medial/superior prefrontal region | Midline/bilateral descriptive channel | Approximate medial prefrontal channel |
| Ch18 | S7–D6 | Right superior/medial | Right superior medial prefrontal region | Right prefrontal ROI | Approximate right superior-medial prefrontal channel |
| Ch19 | S7–D7 | Left superior/medial | Left superior medial prefrontal region | Left prefrontal ROI | Approximate left superior-medial prefrontal channel |
| Ch20 | S8–D5 | Left lateral | Left lateral prefrontal region | Left prefrontal ROI | Approximate left lateral prefrontal channel |
| Ch21 | S8–D7 | Left superior/lateral | Left superior lateral prefrontal region | Left prefrontal ROI | Approximate left superior prefrontal channel |

**Note.** The table was derived from the fNIRS optode montage shown in Figure 1. Blue circles represent light sources and orange squares represent detectors. Source–detector links were identified from the schematic and used to define the measurement channels of the prefrontal montage. Anatomical labels should be interpreted as approximate cortical projections based on the geometry of the standard prefrontal montage, not as participant-specific anatomical coordinates. Individual optode digitization was not performed; therefore, no subject-specific MNI coordinates are reported. Channels were assigned to left or right prefrontal ROIs according to the mediolateral position of each source–detector midpoint. The bilateral prefrontal ROI was calculated as the average of the left and right prefrontal ROIs. The midline channel S7–D4 was retained as a medial/bilateral descriptive channel and should not be interpreted as providing precise localization of a specific medial prefrontal subregion.
